# Anion-specific structure and stability of guanidinium-bound DNA origami

**DOI:** 10.1016/j.csbj.2022.05.037

**Published:** 2022-05-23

**Authors:** Marcel Hanke, Daniel Dornbusch, Christoph Hadlich, Andre Rossberg, Niklas Hansen, Guido Grundmeier, Satoru Tsushima, Adrian Keller, Karim Fahmy

**Affiliations:** a Paderborn University, Technical and Macromolecular Chemistry, Warburger Str. 100, Paderborn 33098, Germany; b Helmholtz-Zentrum Dresden-Rossendorf, Institute of Resource Ecology, Bautzner Landstrasse 400, Dresden 01328, Germany; c Cluster of Excellence Physics of Life, TU Dresden, Dresden 01062, Germany

**Keywords:** DNA origami, Denaturation, Guanidinium, Counteranions, Atomic force microscopy, Circular dichroism

## Abstract

While the folding of DNA into rationally designed DNA origami nanostructures has been studied extensively with the aim of increasing structural diversity and introducing functionality, the fundamental physical and chemical properties of these nanostructures remain largely elusive. Here, we investigate the correlation between atomistic, molecular, nanoscopic, and thermodynamic properties of DNA origami triangles. Using guanidinium (Gdm) as a DNA-stabilizing but potentially also denaturing cation, we explore the dependence of DNA origami stability on the identity of the accompanying anions. The statistical analyses of atomic force microscopy (AFM) images and circular dichroism (CD) spectra reveals that sulfate and chloride exert stabilizing and destabilizing effects, respectively, already below the global melting temperature of the DNA origami triangles. We identify structural transitions during thermal denaturation and show that heat capacity changes Δ*C*_p_ determine the temperature sensitivity of structural damage. The different hydration shells of the anions and their potential to form Gdm^+^ ion pairs in concentrated salt solutions modulate Δ*C*_p_ by altered wetting properties of hydrophobic DNA surface regions as shown by molecular dynamics simulations. The underlying structural changes on the molecular scale become amplified by the large number of structurally coupled DNA segments and thereby find nanoscopic correlations in AFM images.

## Graphical abstract

**Figure:**
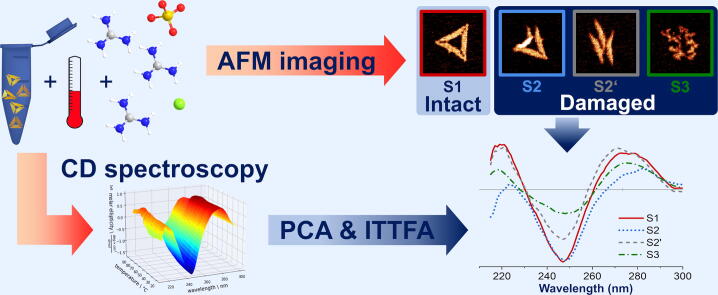

## 1 Introduction

Over the last decade, DNA origami technology [1] has made significant advances and gained more and more relevance in a wide field of applications ranging from biomedicine [2], [3] to biophysics [4], [5] to chemical [6], [7] and synthetic biology [8], [9]. Despite the large number of applications, the unique capabilities of DNA origami to build up biocompatible, well-defined 2D and 3D molecular assemblies of almost arbitrary shape have not been utilized to their fullest extent yet [10]. A widely perceived issue concerns the limited stability of DNA origami nanostructures under conditions that deviate from those employed in DNA origami assembly [2], [11], [12]. Although several DNA origami nanostructures were found to be remarkably stable in various electrolytes featuring different buffers, pH values, and salt compositions [13], [14], [15], as well as during long-term cryostorage [16], [17], the peculiar arrangement of double helices that comprise their individual 3D structure may lead to unexpected and surprising behaviors in denaturing environments such as low-salt conditions [14], [15], under nuclease digestion [15], [18], or in the presence of chaotropes [19]. The latter example is particularly interesting at a fundamental level because the interaction of chaotropic agents such as different guanidinium (Gdm) salts with DNA is highly complex and so far barely understood. Guanidinium chloride (GdmCl), for instance, is a widely employed and potent protein denaturant [20], whose interaction with DNA has only recently received some attention [19], [21], [22], [23]. Moreover, despite having been studied for several decades, even the ubiquitously employed Gdm^+^-induced denaturation of proteins is not completely understood yet [24], [25], [26], [27]. From an application-oriented point of view, the interaction of chemical denaturants with DNA is highly relevant for various processes such as isothermal and low-temperature DNA origami assembly [28], [29], assembly of DNA origami nanostructures from double-stranded (ds) DNA[30] and intact bacteriophages [31], selective DNA origami denaturation for analytical purposes [32], and the removal of DNA origami masks in molecular lithography [33], [34]. In this context, Gdm^+^ is particularly interesting because its effect on DNA origami nanostructures is strongly influenced by concentration, temperature, and the presence and concentration of other ions [19], [23], which may be exploited for fine-tuning its activity to precisely match the requirements of a given application.

**Fig. 1:**
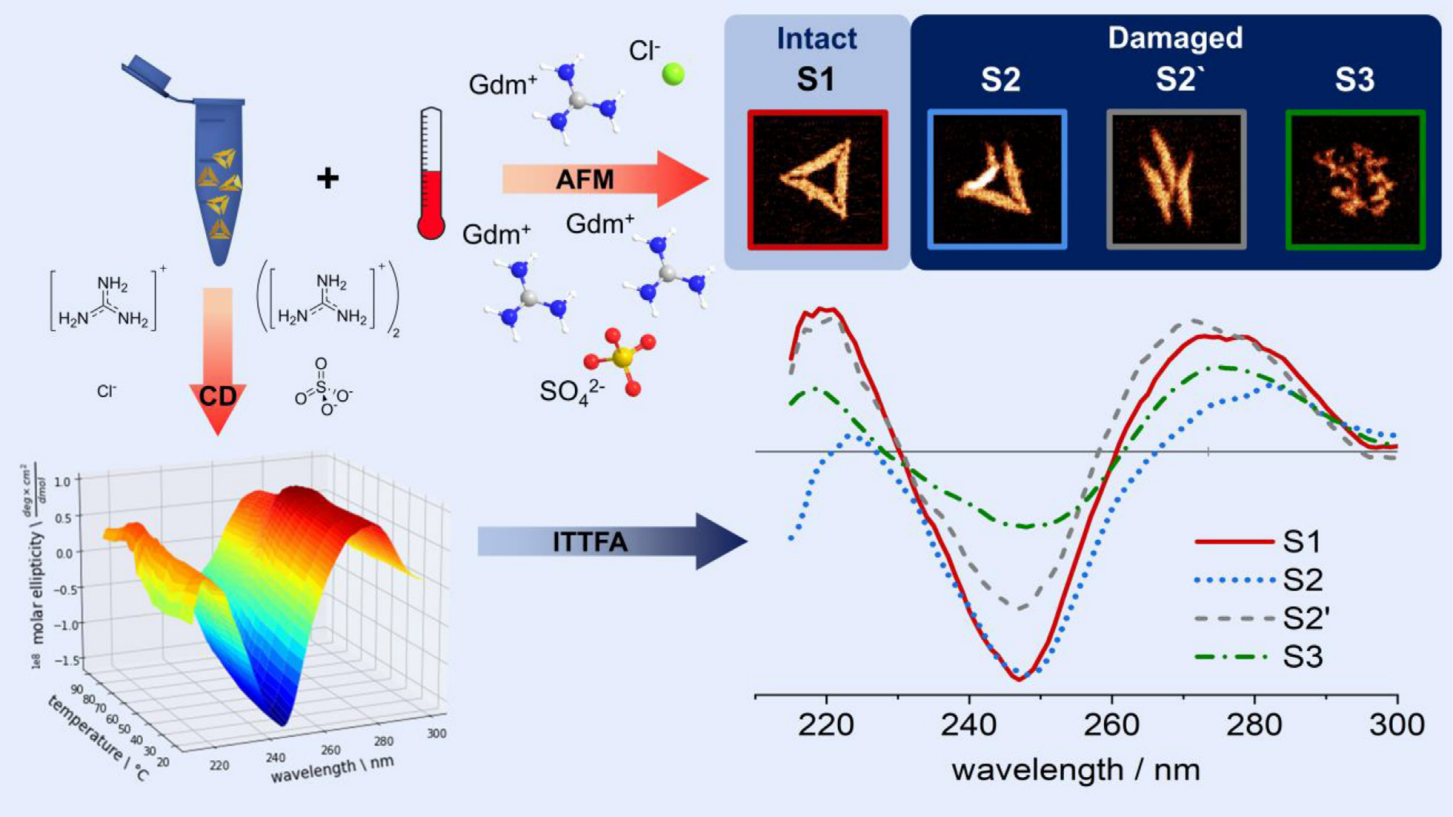
Experimental approach: The nanostructural integrity of DNA origami triangles after exposure to GdmCl and Gdm_2_SO_4_ for one hour is evaluated at selected temperatures by AFM. Temperature-dependent DNA melting in the DNA origami triangles is assessed under equivalent conditions by CD spectroscopy. An iterative target test factor analysis (ITTFA) of the CD spectra primed with the fractions of intact and damaged DNA origami observed by AFM allows us to identify four spectral components assignable to structural states S1, S2, S2′, and S3 (as detailed in the text) occurring during DNA origami melting. The binary AFM classification of intact vs. damaged triangles correlates with CD-spectral features of S1 and S2, respectively. The S2′ and S3 states (pre-melting state and single-stranded DNA at 90 °C, respectively) cannot be clearly identified by AFM. However, selected AFM images of damaged triangles are depicted that may resemble these unresolved states (e.g., S3 is the pure single-stranded scaffold, which when imaged at room temperature exhibits base-paired loops). In the ball-and-stick models, H, C, N, S, O, and Cl atoms are indicated in white, grey, blue, yellow, red, and green, respectively. (For interpretation of the references to color in this figure legend, the reader is referred to the web version of this article.)

The structure of Gdm^+^ (Fig. 1) is characterized by planar hydrophobic faces made up by three NH_2_ groups bound through delocalized bonds to a single sp^2^-hybridized carbon atom. Therefore, hydrophobic interactions as well as the formation of hydrogen bonds between the cation and various amino acids may occur and participate in protein denaturation [24]. Intriguingly, it has been demonstrated that the denaturing effect of the Gdm^+^ ion also depends strongly on its counteranion [24], [25], [26]. In particular, a correlation between the Hofmeister series and the denaturing effect of selective anions has been shown [25]. On the other hand, distortions of the microscopic structure of water are due to the combined effect of the cation-anion pairs, which questions the significance of the Hofmeister concept of “structure maker or breaker” for any single ion [35]. Likewise, direct ion-polymer interactions modulate the detailed energy balance of the denatured vs*.* the native state of a biopolymer in salt solutions [36]. Thus, it cannot be expected that the existing concepts of protein denaturation by Gdm^+^ would equally apply to a complex supramolecular DNA nanostructure. In addition, the present knowledge on the salt-dependence of dsDNA stability has been gained with synthetic or genomic dsDNA of different length, rather than the extended DNA assemblies in DNA origami nanostructures. In contrast to other DNA condensates formed by DNA-condensing molecules, stability of the DNA origami is provided by directed hybridization and not by sequence-independent cation-mediated aggregation. Also in the latter, the analysis of salt effects has naturally focused on cations, which engage in direct electrostatic interactions with the anionic DNA backbone [37], [38].

In this work, we have carried out a thorough analysis of the thermal stability of 2D DNA origami triangles [1] in the presence of either GdmCl or guanidinium sulfate (Gdm_2_SO_4_). In particular, we correlated superstructural changes observed by *ex-situ* atomic force microscopy (AFM) with molecular-level insights obtained by *in-situ* circular dichroism (CD) spectroscopy (Fig. 1). The goal of this analysis was the integration of nanostructural and molecular information into a consistent thermodynamic description of state transitions that underlie thermal DNA origami denaturation in the presence and absence of the Gdm^+^ salts as sketched in Fig. 1. This became possible only by the application of DNA origami nanostructures, whose supramolecular arrangement of close-packed and crosslinked double helices served as an amplifier for subtle steric effects. This physical coupling of nanostructural to molecular information at the helix level not only enabled an optical readout but also provided access to the underlying thermodynamic phenomena.

Our analyses show that the two anions affect structural transitions of the Gdm^+^-bound DNA origami through heat capacity changes, which lead to the hitherto not understood structural damage seen with these Gdm^+^ salts already at ambient temperatures far below the global *T*_m_ of DNA melting. The differently structured hydration shells of chloride and sulfate appear to be pivotal in modulating the energetics of DNA origami conformational transitions by altering the DNA hydration networks.

## 2 Materials and methods

### 2.1 DNA origami synthesis

The synthesis of the DNA origami triangles [1] was based on a previously published protocol [23]. To this end, M13mp18 scaffold (Tilibit) and about 200 staple strands (Eurofins) were mixed at a molar ratio of 1:10 in 10 mM Tris buffer (Sigma-Aldrich) containing 10 mM MgAc_2_ (Sigma-Aldrich). The pH was adjusted to 8.0 with acetic acid. DNA origami assembly was performed during slow cooling from 80 °C to room temperature over 90 min using a Primus 25 advanced thermocycler (PEQLAB). Then, the DNA origami were purified by PEG precipitation. For this purpose, 200 µl of DNA origami solution were diluted in 600 µl Tris/MgAc_2_ buffer and mixed with 800 µl PEG solution containing 1x TAE (Roth), 15 % PEG-8000 (w/v) (Sigma-Aldrich) and 505 mM NaCl (Sigma-Aldrich). This solution was centrifuged using a VWR microcentrifuge at 14,000 rcf for 30 min at 18 °C, after which the supernatant was carefully removed with a pipette. The precipitate was re-dissolved in about 30 µl Tris/MgAc_2_ buffer overnight. The DNA origami concentration of the resulting solution was determined using an Implen Nanophotometer P330 and adjusted to 100 nM with Tris/MgAc_2_ buffer.

### 2.2 AFM imaging and analysis

GdmCl solution (8 M) and Gdm_2_SO_4_ salt were purchased from Sigma-Aldrich. Gdm_2_SO_4_ salt was dissolved in HPLC-grade water (VWR) to reach a concentration of 8 M. For each experiment, stock solutions of GdmCl and Gdm_2_SO_4_ were mixed with Tris/MgAc_2_ buffer and DNA origami triangle stock solution to reach final Gdm^+^ concentrations of 1 M, 2 M, 4 M, and 6 M for GdmCl and 2 M, 4 M, 8 M, and 12 M for Gdm_2_SO_4_, respectively, at a constant DNA origami concentration of 5 nM. 100 µl samples of the resulting solutions were incubated for 1 h at different temperatures (23 °C, 30 °C, 37 °C, 42 °C) using a Primus 25 advanced thermocycler.

After 1 h of incubation, 1 µl of sample solution was deposited on freshly cleaved mica, covered with 50 µl of Tris/MgAc_2_ buffer, and incubated for 5 min. Then, the sample was rinsed with about 6 ml of HPLC-grade water and dried in a stream of ultra-pure air. AFM imaging was performed in air using a Bruker Dimension ICON in ScanAsyst PeakForce Tapping mode with ScanAsyst-Air cantilevers (Bruker) or an Agilent 5100 in intermittent contact mode with HQ:NSC18/Al BS cantilevers (MikroMasch).

For the statistical analyses, approx. 300–600 DNA origami nanostructures from at least three AFM images taken at different positions on the surfaces of up to three independent samples were analyzed for each experimental condition using Adobe Photoshop software. The DNA origami nanostructures visible in the AFM images were classified either as intact or damaged based on visual evaluation of their shape as previously described [14], [39]. In particular, any DNA origami shape that visibly deviated from a perfectly assembled triangle was considered damaged, even if the deviation was comparably small such as a partially ruptured vertex. No distinction was made regarding the thermodynamic states S2, S2′, and S3, because (i) these states are not well defined at the nanostructure level and (ii) AFM imaging was performed at room temperature, where S2′ is populated to less than 12% and S3 entirely absent (Fig. 8). The relative fractions of intact and damaged DNA origami nanostructures were determined by manually counting the absolute numbers of each species visible in each AFM image. The so determined fractions per AFM image were then averaged over at least three AFM images per condition. Fractions are presented as mean values with standard deviations as error bars.

### 2.3 CD spectroscopy

DNA origami solutions (56 µl, 100 nM) were mixed with Gdm^+^ salt stock solutions and Tris/MgAc_2_ buffer to a total of 140 µl and a total concentration between 30 and 40 nM DNA origami triangles, 10 mM Tris and 10 mM MgAc_2_. The final concentrations of Gdm^+^ salts were 0.5 M, 1 M, 2 M, 4 M and 6 M at a constant DNA origami concentration of 40 nM. These solutions were placed in a quartz cuvette (Helma) with a cell length of 1 mm and measured immediately. CD measurements were performed using a Jasco 815 CD spectrometer with a heat denaturation protocol from 20 °C to 90 °C with a temperature ramp of 5 °C/min (three spectra were co-added at 20 °C, 30 °C, 40 °C, 45 °C, 50 °C, 53 °C, 56 °C, 58 °C, 60 °C, 62 °C, 64 °C, 67 °C, 70 °C, 75 °C, 80 °C, 90 °C). Spectra were recorded from 330 nm to 200 nm with a data pitch of 1 nm, a scanning speed of 100 nm/min, a bandwidth of 3 nm, and a digital integration time (D.I.T.) of 2 sec.

### 2.4 Pre-treatment of the CD data and calculation of melting temperatures

The threshold for the CD data was set at a high tension (HT) voltage of 600 V. The concentration of DNA origami was calculated from the absorption at 260 nm at 20 °C using the Beer-Lambert law with an optical density of 1 corresponding to 0.2 µg/ml per double-strand base pair. To illustrate the behavior of the CD spectra, the changes in CD values were correlated with those of temperature for windows of three measurement points each [40], effectively showing the first derivative. The absorption values at 260 nm were used to calculate the melting temperature from the hypochromic shift [41]. Therefore, we fitted a higher and lower baseline and determined the intersection of their median with the melting curve approximated with linear slopes between nearest two points. The normalized absorption values between zero (initial baseline) and one (final baseline) were taken as the single-stranded (ss) DNA fraction and the melting temperature *T*_m_ was determined from the interpolated data at a fraction of 50 % ssDNA.

### 2.5 Principal component analysis (PCA) and iterative target test factor analysis (ITTFA)

For investigating components we used the program ITTFA [42]. First, we analyzed the eigenvalues of the covalent data matrix and the given relative uncertainties from the PCA. Then, ITTFA for three and four components was performed using the fraction of ssDNA to fix one component (S3). The fraction of the intact DNA origami triangles (S1) from the AFM measurement was used in the 23 to 45 °C range for the component S1.

### 2.6 Thermodynamic modelling

The free enthalpy differences between hypothetical structural states were expressed as.

(1) ΔG=ΔH-TΔS

with

(2) $$\Delta H=\Delta H_{0}+\Delta C_{p}\left(T-T_{0}\right)$$

and

(3) $$\Delta S=\Delta S_{0}+\Delta C_{p}T\ln\left(T/T_{0}\right)$$

*T*_0_ is a reference temperature at which two hypothetical states are equally populated. The parameters entropy and enthalpy change Δ*S* and Δ*H*, respectively, as well as the heat capacity change Δ*C*_p,_ were varied using LabTalk in Origin to approximate the temperature dependence of the principal components. The thermodynamically determined curves were then used again in PCA to obtain model-conform component spectra. The process was iterated for the Gdm_2_SO_4_ and GdmCl data sets to increase spectral similarity between the respective S1, S2, S2′, S3 spectra. The final component spectra (Fig. S6c) are the averages of the component spectra from the Gdm_2_SO_4_ and GdmCl PCA. Thereby, a common spectral basis was used for both data sets. It complied with the thermodynamic parameters in Table 1 with which it reproduced the salient traits of the temperature-dependent CD spectra (Fig. S4).

**Table 1:** Thermodynamic parameters for state transitions in Gdm^+^-bound DNA origami triangles at 4 M Gdm^+^.

Anion	S1 → S2		S1 → S2′		S2 → S3	
	SO_4_^2−^	Cl^−^	SO_4_^2−^	Cl^−^	SO_4_^2−^	Cl^−^
Δ*H* (kJ)	150	120	211	360	550	480
Δ*S* (kJ/K)	3.947	3.296	0.661	1.137	8.620	8.247
Δ*C*_p_ (kJ/K)	1.800	16.500	5.000	13.000	(20.000)	(10.000)
*T*_0_ (°C)^a)^	38.0	36.4	46	43.6	63.8	58.2

^a)^*T*_0_: temperature at which Δ*H* and Δ*S* are defined and at which the indicated structural states become equally populated (crossing points of the curves in Figs. 8 and S6a).

### 2.7 MD simulations

Molecular dynamics (MD) simulations and data analyses were performed using AMBER 15 program package [43] with OL15 force field applied on DNA. For Mg^2+^ ions [44], additional parameters were employed. MD parameters of the Gdm^+^ and SO_4_^2−^ ions were obtained by means of antechamber module of AMBER package utilizing Gaussian 16 quantum chemistry program package [45] for calculating partial charges. For GdmCl, simulation box contains 80 Gdm^+^–Cl^−^ pairs as well as 725 waters whereas for Gdm_2_SO_4_ the simulation box contains 80 Gdm^+^, 40 SO_4_^2−^, and 725 waters. For the DNA system, the Drew-Dickerson B-DNA dodecamer was used for which Mg^2+^ ions have been added to compensate the negative charge of DNA. 500 steps of steepest decent and 500 steps of conjugate gradient with 500 kcal mol^−1^ Å^−1^ harmonic restraint on the DNA were initially conducted after which 1000 steps of steepest decent and 1500 steps of conjugate gradient were performed without constraints. 40 ps of heating of the system from 0 to 300 K with 10 kcal mol^−1^ Å^−1^ harmonic restraint on the DNA, after which another 1 ns preconditioning run was performed at 300 K without restraint on the solutes. Finally, 20 ns (for Gdm^+^ salts) and 50 ns (for DNA with Mg^2+^ counterions) MD run was performed in a periodic boundary condition in NPT ensemble [46]. Simulations were terminated and restarted every 5 ns to avoid artificial convergence to particular geometries. The SHAKE algorithm, a 2 fs time integration step, 10 Å cutoff for non-bonded interactions, and the particle mesh Ewald (PME) method were used. MD trajectories were recorded at each 50 ps.

## 3 Results

### 3.1 AFM imaging

We investigated the interaction of triangular DNA origami nanostructures with the two selected Gdm^+^ salts at the nanostructure level by ex-situ AFM. We specifically chose ex-situ AFM characterization under dry conditions for two reasons. (i) Initial in-situ AFM imaging experiments revealed that the presence of high concentrations of Gdm_2_SO_4_ not only suppresses DNA origami adsorption at the mica surface but can even lead to the desorption of once-adsorbed DNA origami (Fig. S1). Therefore, in-situ AFM imaging is not possible under all buffer conditions investigated in the present work. (ii) Previous work has shown that DNA origami denaturation in GdmCl may proceed over several hours [19]. In order to ensure identical incubation times for all samples and to freeze the state of degradation obtained at a certain time point, the reaction thus needs to be stopped after immobilization of the DNA origami nanostructures at the mica surface by washing and removal of residual Gdm^+^. Therefore, to avoid such issues, the DNA origami triangles were incubated with different concentrations (1 – 6 M) of the respective salt for 1 h at different temperatures ranging from 23 to 42 °C. After dilution in Gdm^+^-free buffer to facilitate efficient adsorption, the samples were incubated on mica surfaces for 5 min, gently washed, and dried. Then, AFM images were recorded in the dry state to evaluate the relative fractions of “intact” and “damaged” DNA origami. Three examples of DNA origami triangles categorized as “damaged” are shown in Fig. 1 (AFM images labeled S2, S2’, and S3). This category includes any deviation from the perfect triangular shape of untreated DNA origami nanostructures (Fig. 1, AFM image S1), ranging from ruptured vertices to completely denatured structures that feature only the scaffold.

**Fig. 2:**
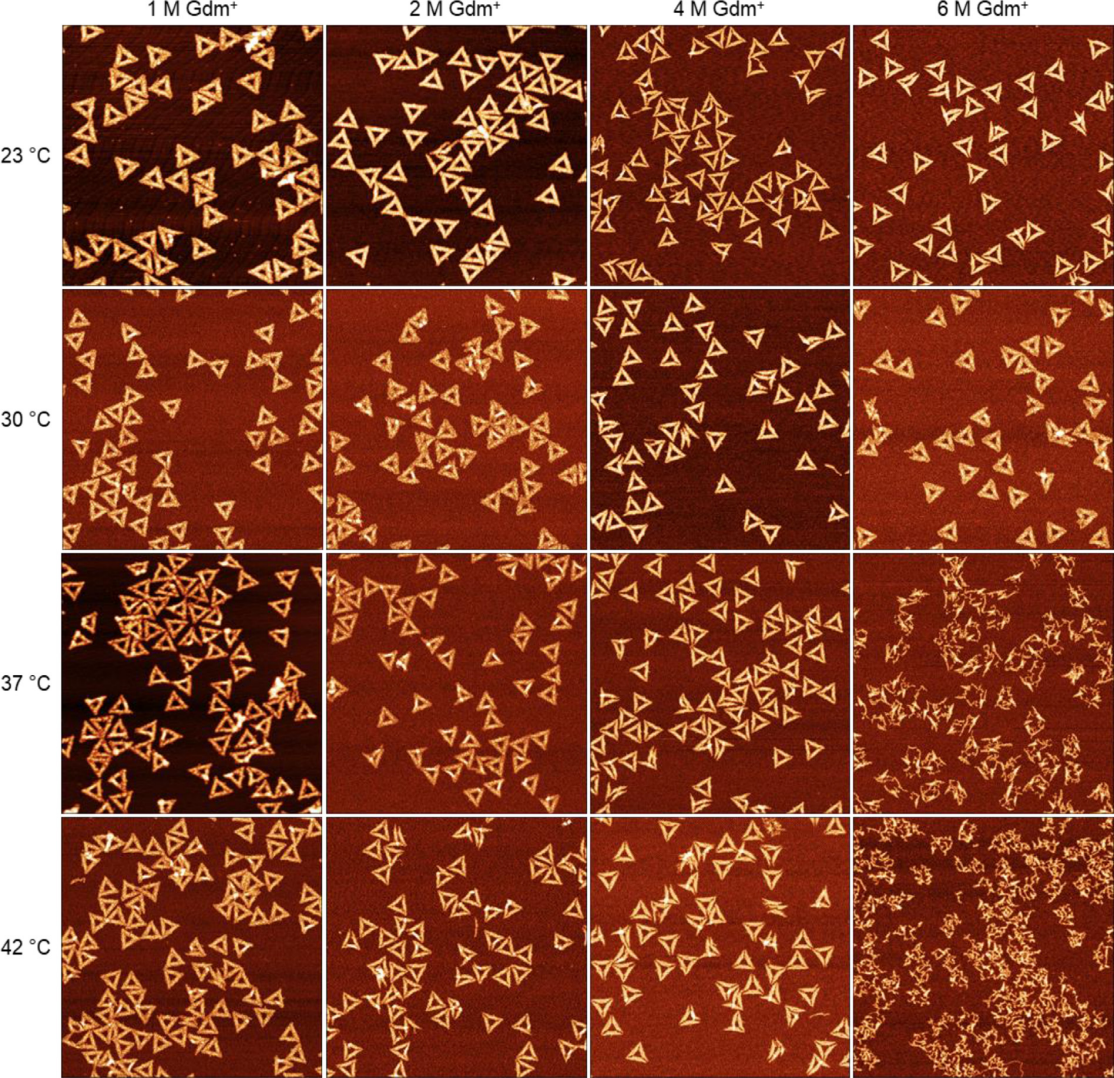
AFM images of DNA origami triangles deposited on mica after 1 h incubation in GdmCl at different Gdm^+^ concentrations and temperature conditions. All images have a size of 1.5×1.5 µm^2^. The color range was set to automatic with tails cut off.

The general effect of GdmCl on the DNA origami triangles has been characterized previously [19]. However, we have employed electrolyte conditions in the present work that have been matched with CD spectroscopic conditions (10 mM Tris acetate, pH 8.0, with 10 mM MgAc_2_ instead of 40 mM Tris acetate, pH 8.5, with 1 mM EDTA and 10 mM MgCl_2_ as used previously) to enable a thorough correlation of superstructural transitions with molecular details. The overall trend (Fig. 2) is very similar to the previously reported one, showing intact triangular shapes between 23 and 42 °C for GdmCl concentrations up to 4 M. At 6 M GdmCl, intact triangles are observed only below 37 °C, while higher temperatures result in the complete denaturation of all DNA origami nanostructures.

**Fig. 3:**
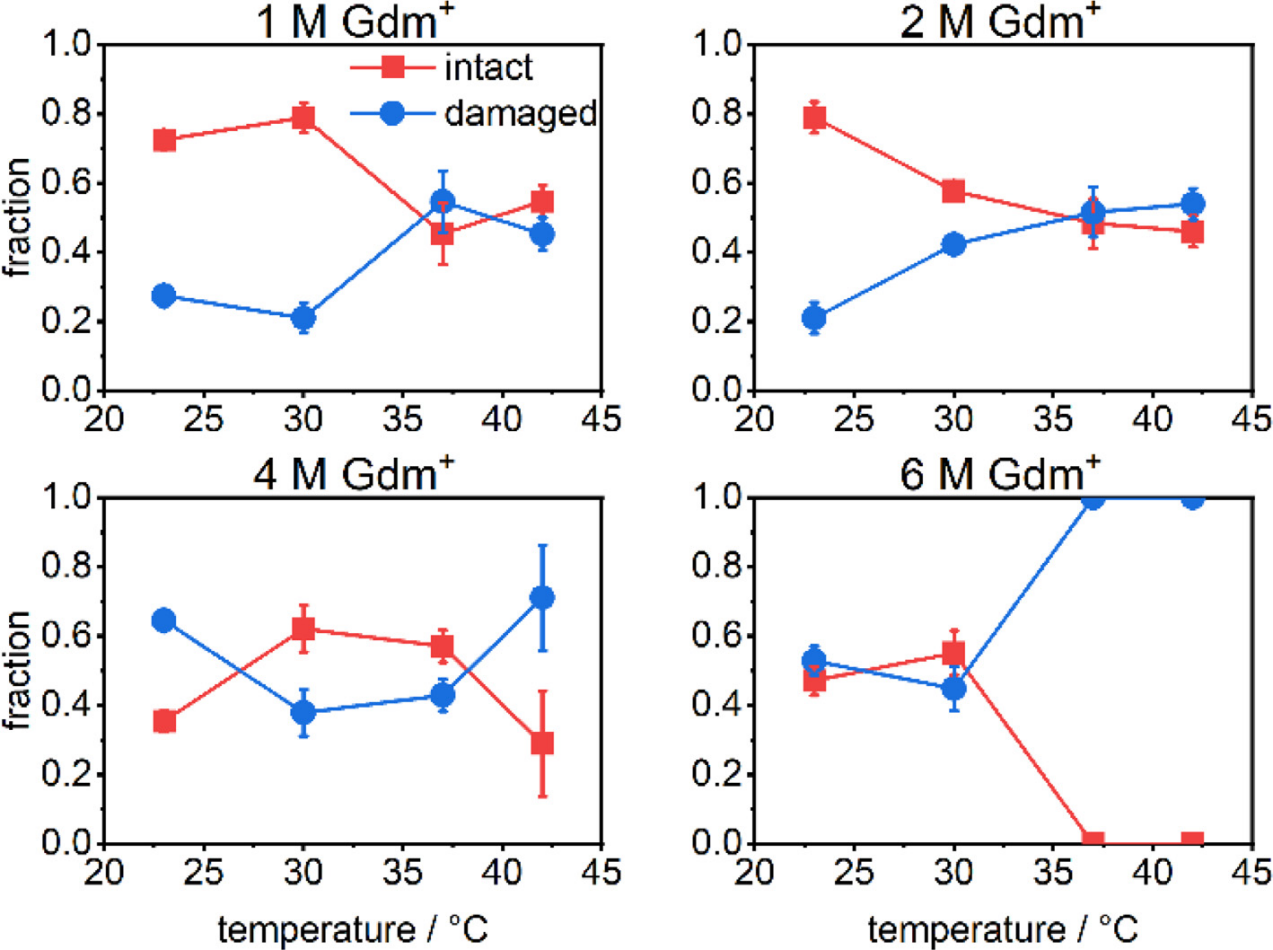
Mean fractions of intact and damaged DNA origami triangles after 1 h incubation in GdmCl at different Gdm^+^ concentrations and incubation temperatures. Values represent averages over at least three AFM images with standard deviations given as error bars.

The statistical analysis of the AFM images shown in Fig. 3 reveals that in 1 M GdmCl at 23 °C, the fraction of intact DNA origami is slightly above 70 % and thus lower than typical assembly yields of about 90 % observed in the absence of chaotropic agents [16]. This fraction barely changes upon increasing the temperature to 30 °C but drops to about 50 % at 37 and 42 °C. The corresponding AFM images in Fig. 2 show that the connections between two trapezoids are severed in some of the damaged DNA origami, suggesting that the DNA origami triangle responds most sensitively to Gdm^+^ denaturation at the vertices. This particular sensitivity of the vertices has been observed previously and attributed to the short length of the bridging staples, which are the shortest in the whole triangle design and thus have particularly low melting temperatures [19], [47].

Increasing the GdmCl concentration to 2 M does not affect the DNA origami stability at 23 °C, whereas at 30 °C, only about 60 % of intact structures prevail and higher temperatures further reduce this fraction to below 50 % at 42 °C. The corresponding AFM images in Fig. 2 reveal preferential damage at the vertices similar to that in 1 M GdmCl. However, some DNA origami triangles are ruptured at all three vertices, resulting in a loosely connected assembly of largely intact trapezoids.

In case of 4 M GdmCl, the results of the statistical analysis reveal a counter-intuitive temperature dependence of the DNA origami stability. Already at 23 °C, most of DNA origami exhibit ruptured vertices or broken trapezoids (see the corresponding AFM image in Fig. 2), and only about 35 % of the DNA origami remain intact. Surprisingly, the fraction of intact DNA origami triangles recovers to about 60 % upon increasing the temperature to 30 °C. The intact fraction persists at 37 °C with a percentage comparable to that observed in 2 M GdmCl. At 42 °C, the fraction of intact DNA origami nanostructures drops again to about 30 %. At first glance, this behavior suggests that DNA origami nanostructures partially denature at low temperature and subsequently re-establish their intramolecular interactions and thereby recover their shape at elevated temperature.

In 6 M GdmCl, about half of the DNA origami nanostructures stay intact between 23 and 30 °C. At 37 °C, however, the damage drastically increases with intact DNA origami being virtually absent. Notably, the DNA origami morphology under these conditions does not show any resemblance with the original triangular shape anymore but rather appears mostly melted. At 42 °C, any remaining indications of the original triangular superstructure have disappeared completely.

**Fig. 4:**
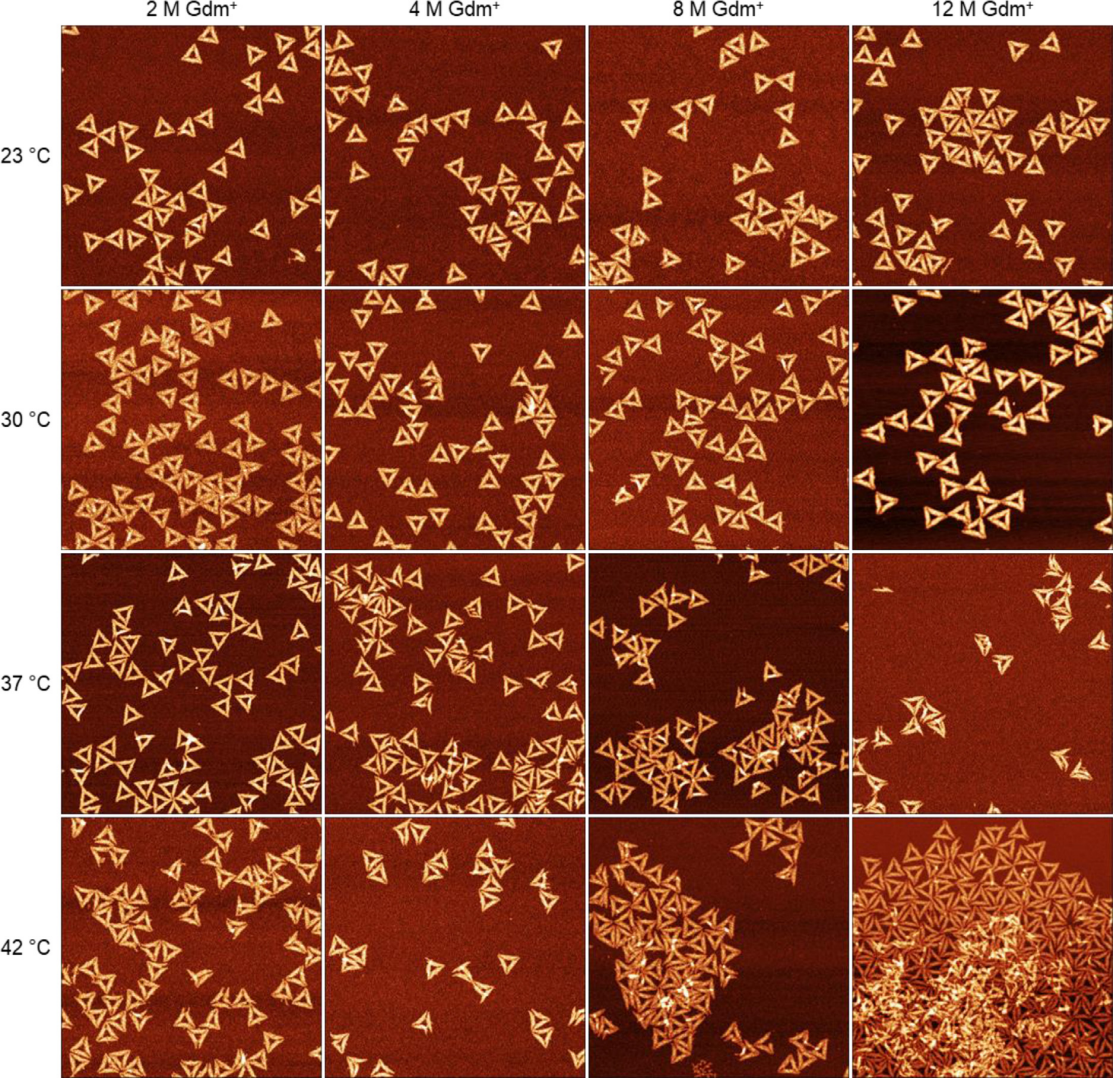
AFM images of DNA origami triangles deposited on mica after 1 h incubation in Gdm_2_SO_4_ at different Gdm^+^ concentrations and temperature conditions. All images have a size of 1.5×1.5 µm^2^. The color range was set to automatic with tails cut off.

**Fig. 5:**
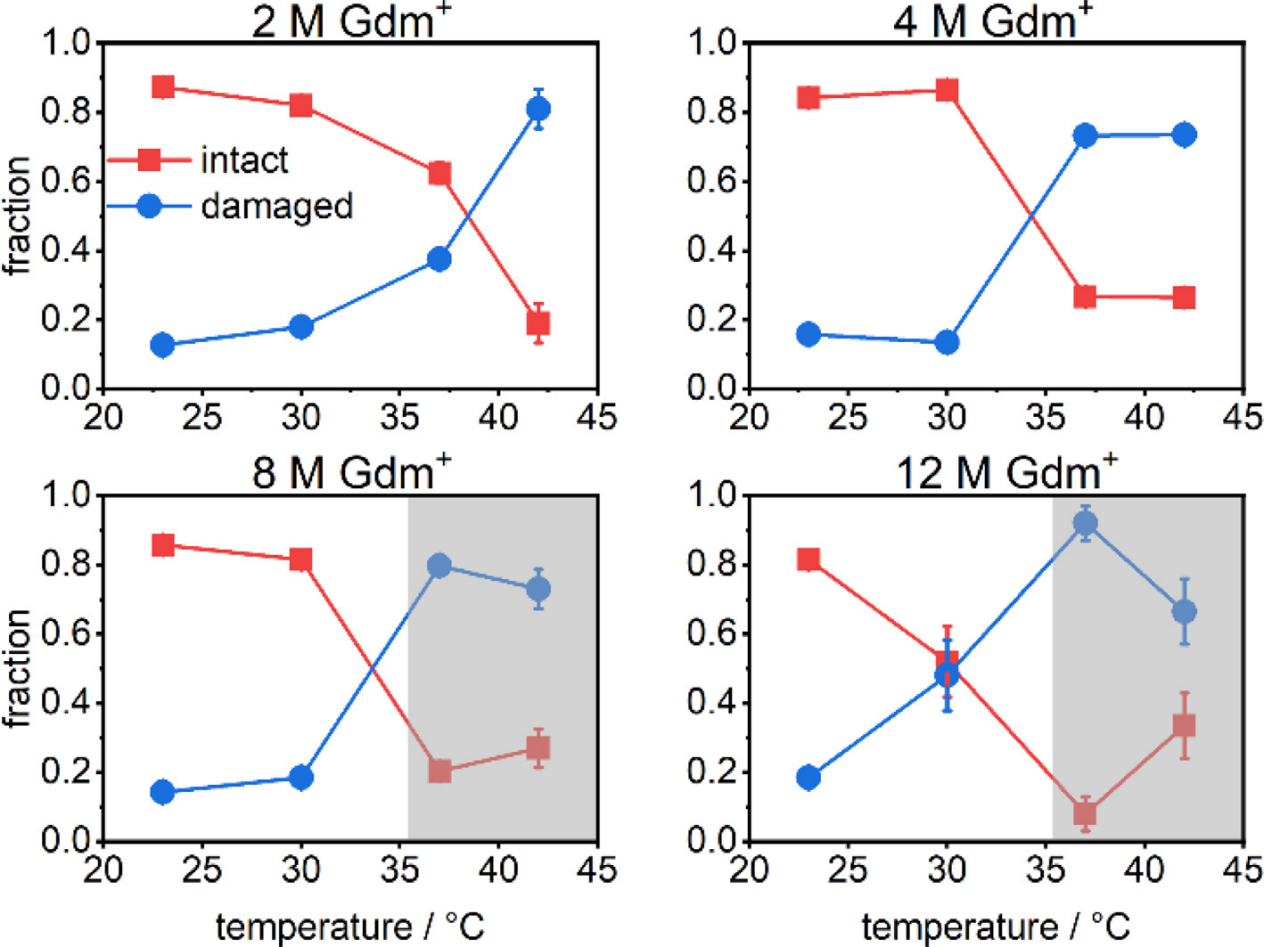
Mean fractions of intact and damaged DNA origami triangles after 1 h incubation in Gdm_2_SO_4_ at different Gdm^+^ concentrations and incubation temperatures. Values represent averages over at least three AFM images with standard deviations given as error bars. The shaded areas indicate the occurrence of DNA origami clustering.

While GdmCl clearly exerts denaturing effects on the DNA origami, the influence of Gdm_2_SO_4_ is markedly different. Sulfate anions have been shown to a stabilize proteins [25] and an analogous stabilizing effect is also seen for the DNA origami triangles in Fig. 4. However, the overall situation appears surprisingly complex. In 2 M Gdm^+^, corresponding to 1 M Gdm_2_SO_4_, more than 80 % of the DNA origami nanostructures are structurally intact at 23 °C and 30 °C (Fig. 5). At 37 °C, however, the fraction of intact DNA origami decreases to about 62 %. Upon increasing the temperature further to 42 °C, the fraction of intact DNA origami nanostructures drops to only about 18 %. This is in stark contrast to the behavior seen for GdmCl in Fig. 3, where the fraction of intact DNA origami in 2 M GdmCl never drops below 40 %. This illustrates impressively the importance of the counteranions in modulating the efficiency of Gdm^+^-induced DNA origami denaturation. However, despite this astonishingly small fraction of intact DNA origami, the corresponding AFM image in Fig. 4 shows surprisingly little damage to the individual DNA origami nanostructures, which is mostly comprised of disrupted vertices and partially damaged trapezoids resulting in an overall deformation of the triangular shape.

For Gdm_2_SO_4_ at 4 M Gdm^+^, the denaturation at intermediate temperatures becomes more pronounced. At 23 °C and 30 °C, about 85 % intact DNA origami nanostructures are observed, whereas this value decreases to 25 % at 37 °C. Increasing the temperature to 42 °C does not result in a further decrease of intact DNA origami. However, the corresponding AFM images in Fig. 4 reveal a slight depletion of DNA origami adsorbed at the mica surface.

At a Gdm^+^ concentration of 8 M, the observed damage is similar to that of 4 M. More than 80 % of the DNA origami nanostructures are intact at 23 °C and 30 °C, while at 37 °C and 42 °C, this value drops to about 20 % and 28 %, respectively. Strikingly, the original triangular shape can still be identified for all DNA origami nanostructures and no DNA origami melting is observed at all (Fig. 4). Even at 12 M Gdm^+^, more than 80 % of the DNA origami remain intact at 23 °C. This is again in striking contrast to the case of 6 M GdmCl, where 50 % of the DNA origami are damaged at this temperature (Fig. 3). At 37 °C, the fraction of intact DNA origami nanostructures drops to less than 10 %. Surprisingly, at 42 °C, this value recovers to about 30 %.

Close evaluation of the AFM images recorded for Gdm_2_SO_4_ at 8 M and 12 M Gdm^+^ in Fig. 4 reveals that the DNA origami nanostructures show a tendency to form clusters at the mica surface. This clustering is getting more pronounced with increasing temperature, resulting in large agglomerates at 37 °C and 42 °C, respectively. Intriguingly, the observed clusters seem to consist mostly of intact DNA origami. However, overlapping and multilayer formation makes it very challenging to identify and count the damaged and intact DNA origami triangles inside the clusters, so that the corresponding fractions given in Fig. 5 (shaded areas), may over- or underestimate either species since they are mostly based on isolated DNA origami outside the clusters. This tendency of the DNA origami nanostructures to form clusters in Gdm_2_SO_4_ probably results from the salting-out effect of the kosmotropic SO_4_^2−^ anions [48]. Salting-out of biomolecules occurs when added salt ions neutralize charges at the biomolecule surface and dehydrate hydrophobic surface patches, thereby triggering biomolecular aggregation and precipitation. Kosmotropic ions such as SO_4_^2−^ are particularly efficient in this regard and it was recently shown that also DNA origami nanostructures can be salted out by high concentrations of (NH_4_)_2_SO_4_ [48].

### 3.2 CD spectroscopy

**Fig. 6:**
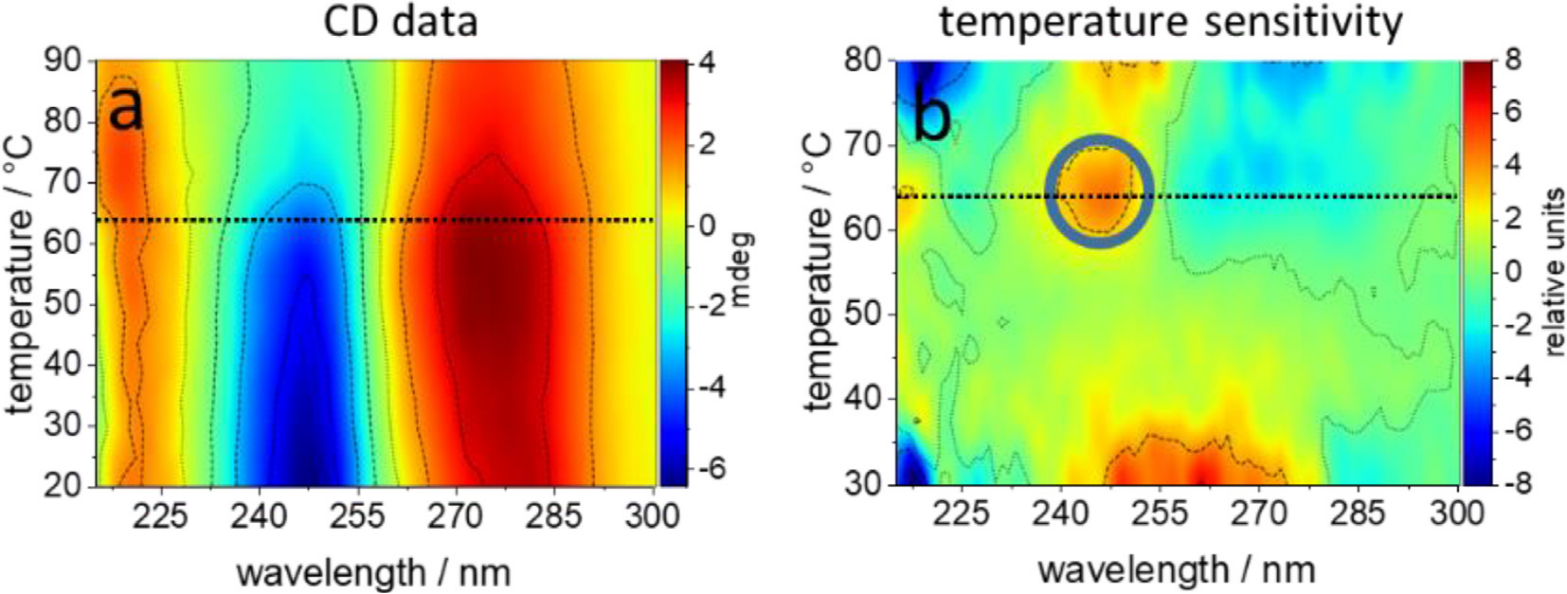
CD amplitudes of DNA origami (31 nM) during thermal denaturation in the absence of Gdm^+^. (a) The maximal and minimal CD signals reach 4.1 and −6.4 mdeg, respectively. (b) Temperature sensitivity plot of the DNA origami in the absence of Gdm^+^. Two-dimensional correlation of the CD spectra (as in a) with temperature is based on the comparison of three consecutive temperature-dependent spectra as described [40] and reveals the spectral regions with high (yellow to red) relative temperature sensitivity. As an orientation: the maximal absolute temperature sensitivity of the CD signal at 247 and 275 nm is + 0.33 mdeg/K, and −0.12 mdeg/K, respectively, when calculated conventionally from the derivative of the CD signals with respect to temperature. The dotted lines mark the global melting temperature of *T*_m_ = 63.9 °C determined from the 260 nm absorption. (For interpretation of the references to color in this figure legend, the reader is referred to the web version of this article.)

We have attempted to correlate the nanostructural changes seen in the AFM images with molecular features that can be addressed by CD spectroscopy and UV absorption, such as chirality and base pair opening, respectively. Fig. 6a shows the color-coded CD amplitudes of the DNA origami during thermal denaturation in the absence of Gdm^+^. The hallmarks of the CD signature of DNA are the 240 – 250 nm (negative) and 265 – 280 nm (positive) lobes colored in dark blue and dark red, respectively. The information content of the rather complex spectral changes was analyzed by 2D correlation. Thereby, the CD signal at a single wavelength can be correlated directly with the change of temperature resulting in a temperature-sensitivity (TS) plot, which identifies changes in the CD amplitude similar to a first derivative of the CD traces with respect to the temperature (Fig. 6b). The prominent region around 247 nm and 62 – 65 °C in the TS plot shows that the negative CD signal loses its intensity most pronouncedly at this temperature. The midpoint temperature (*T*_m_) of DNA melting (monitored simultaneously by the hyperchromic effect at 260 nm absorption, Fig. S5) is plotted as a horizontal line through the TS plot. Its intersection with the 247 nm peak evidences that the loss of chirality upon base unstacking occurred concomitantly with base pair opening at a common *T*_m_ of 63.9 °C, as highlighted by the encircled region of the plot.

**Fig. 7:**
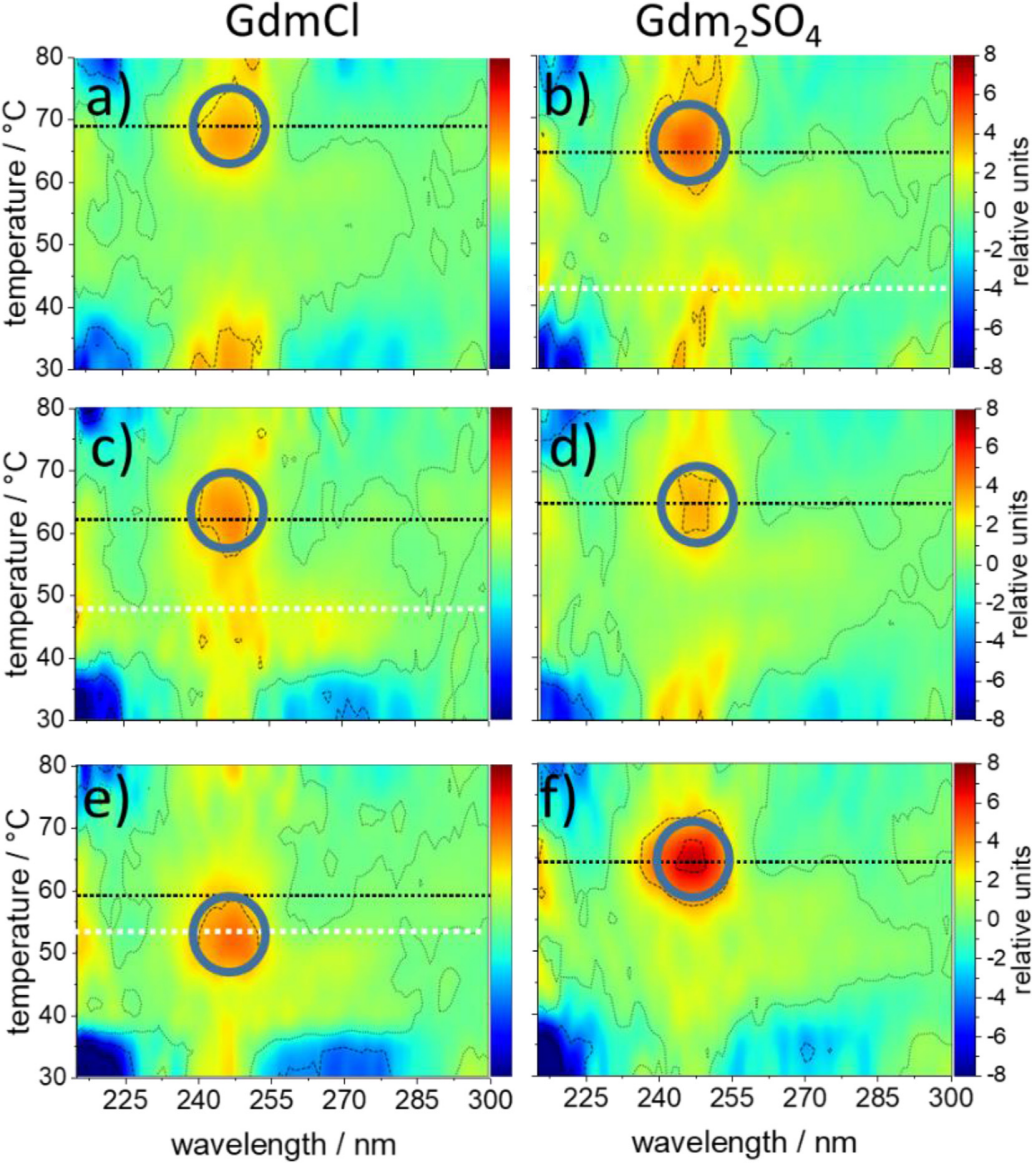
Temperature sensitivity plots of thermal denaturation of DNA origami in the presence of Gdm^+^ at concentrations of 1 M (a, b) 2 M (c, d) and 4 M (e, f).The counteranion was Cl^−^ (left) or SO_4_^2−^ (right) at the indicated Gdm^+^ concentrations. Dotted lines mark the global *T*_m_ in black and the conformational transition CT-2 (∼47 °C) in white. Circles locate the maximal temperature sensitivity of the CD signal around 247 nm at the global *T*_m_. In 4 M Gdm^+^, this correlation is preserved with SO_4_^2−^ but lost with Cl^−^ (e, f). All plots are presented in the same relative scale (given on the right hand panels) and were generated as referenced in Fig. 6b.

The influence of the counteranions Cl^−^ and SO_4_^2−^ on the thermal melting of the DNA origami in the presence of Gdm^+^ can be assessed from the TS plots shown in Fig. 7. In 1 M Gdm^+^, the maximum unstacking transition in the presence of Cl^−^ coincides again with maximum base pair opening at a *T*_m_ of 65.7 °C, *i.e.*, 4 °C higher than in the control. Additionally, GdmCl inverts the TS of the CD signal between 260 and 280 nm (compare color codes of Fig. 6b and Fig. 7a) upon heating from 20 °C to 40 °C, where the control shows an increase of the positive CD lobe. The features induced by GdmCl correspond to a reduction of the underlying CD signal. Likewise, the CD signal increases between 240 and 250 nm, such that also the negative CD lobe becomes weaker within the same temperature range. As a consequence, overall chirality decreases upon a rise in temperature from 23 to 40 °C. We assign these features to a first conformational transition (CT-1) which occurs far below the global *T*_m_ of the entire DNA origami. At the same Gdm^+^ concentration of 1 M, the features of CT-1 are qualitatively reproduced with Gdm_2_SO_4_ (Fig. 7b), suggesting that Gdm^+^ is primarily responsible for CT-1 below 40 °C. However, additional temperature-induced CD-spectral changes occur at about 45 °C in Gdm_2_SO_4_ (white dotted line). The underlying spectral phenotype corresponds to a reduction of the negative CD lobe at 247 nm and a slight increase of the positive lobe between 260 and 270 nm upon heating from 20 to 40 °C. We assign this to a second conformational transition CT-2. It is observed for GdmCl only at 2 M Gdm^+^ (Fig. 7c), showing that SO_4_^2−^ is about twice as efficient as Cl^−^ in inducing CT-2.

**Fig. 8:**
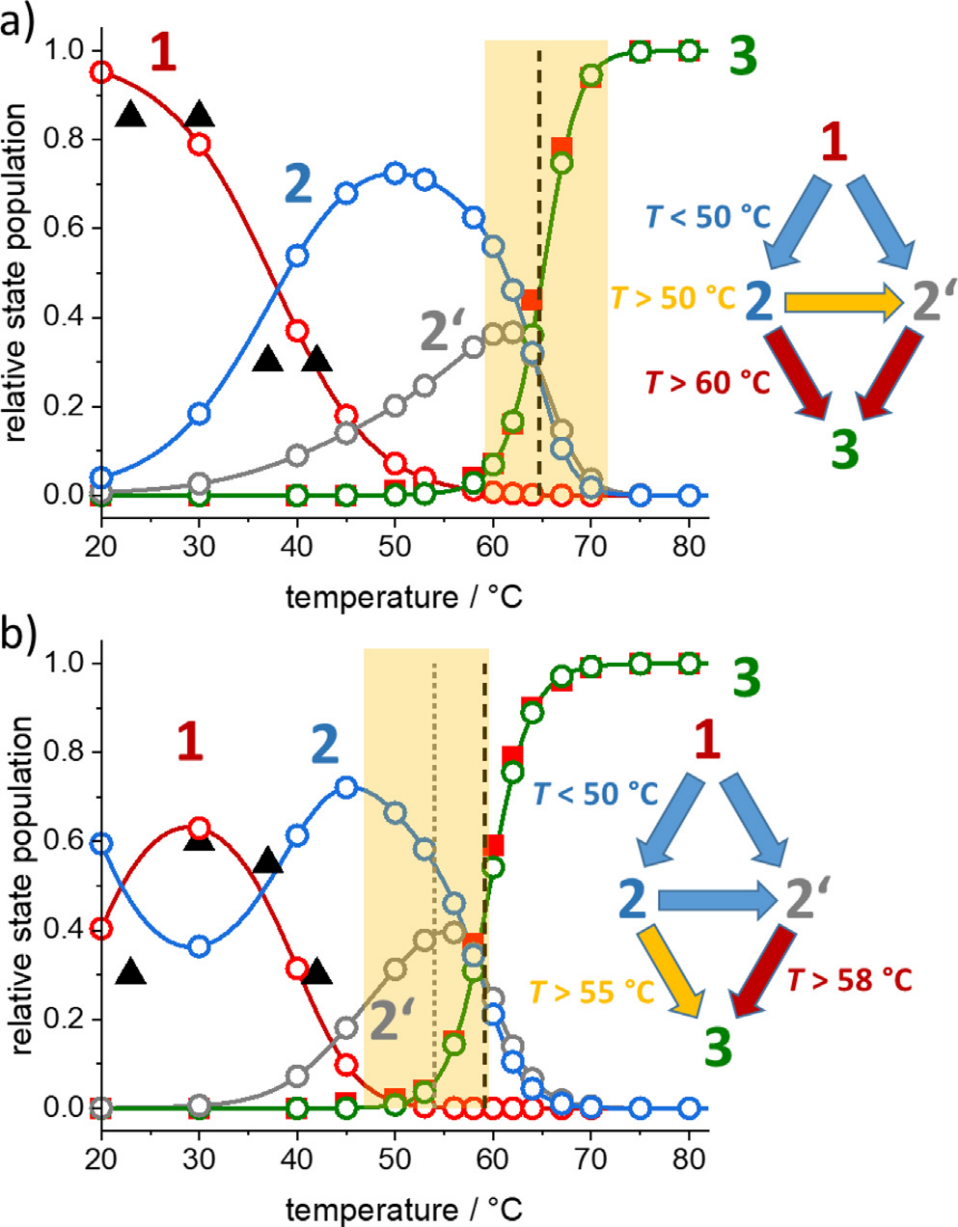
Principal component analysis and thermodynamic modelling of the thermal denaturation of Gdm^+^-bound DNA origami triangles. Sets of sixteen temperature-dependent CD spectra recorded at 4 M Gdm^+^ were decomposed into four principal components for the Gdm_2_SO_4_ (a) and GdmCl (b) solutions. The components one (red) and two (blue), respectively, with “intact” (S1) and “damaged” (S2) populations seen in the AFM images below 45 °C (black triangles). Component S3 (green) follows the 260 nm UV absorption (red filled squares) monitoring ssDNA. Component S2’ (gray) was not restricted and represents a pre-melting intermediate. Circles show the population of the respective states used for the generation of the CD spectra (Fig. S6) of each component by PCA. Solid lines are the results of a thermodynamic model, with the parameters in Table 1. The insets emphasize the predominant state transitions in the indicated temperature ranges. Red filled squares: normalized 260 nm absorption change. (For interpretation of the references to color in this figure legend, the reader is referred to the web version of this article.)

Above 2 M Gdm^+^, the structural impact of the anions on the DNA origami differs fundamentally. In 4 M GdmCl, CT-1 becomes stronger and the CT-2 below 50 °C disappears (Fig. 7e). SO_4_^2−^ abolishes CT-2 already at 2 M Gdm^+^, in line with the stronger effect of SO_4_^2−^ compared to Cl^−^ on the otherwise very similar spectral patterns produced by the Gdm^+^ salts. However, the main unstacking transition in 4 M GdmCl appears already at 53 °C, *i.e.*, about 6 °C below the *T*_m_ of 59.4 °C for base pair opening, whereas at the same Gdm^+^ concentration, SO_4_^2−^ affects neither the dephasing of base unstacking and base pair opening nor the global *T*_m_ of 64.7 °C (Fig. 7f).

The deviation of the *T*_m_ of base pair opening from that of the main unstacking transition is the most prominent and least expected anion-specific effect of the tested Gdm^+^ salts on the global melting of the DNA origami triangles. The underlying anion-specific mechanisms are probably also responsible for the different effects of the salts on the stability of the triangles revealed by AFM already below the global *T*_m_.

Therefore, CD-spectral signatures and relative concentrations of putative structural components underlying the denaturation between 20 °C and 90 °C were further evaluated by Principal Component Analysis (PCA). In order to provide structurally interpretable results from this inherently ambiguous method, we have imposed the following constraints on the PCA. The “intact” and “damaged” fractions of the DNA origami where assigned to components S1 and S2, respectively, such that their relative concentrations could be narrowed in for the four temperatures at which AFM imaging was performed. Furthermore, component S3 was determined from the normalized 260 nm absorption, thus correlating with the amount of ssDNA. With these restrictions, it was possible to decompose the CD spectra into four temperature-dependent structural states with the fourth component S2’ maintaining the total DNA concentration at unity. The component spectra of the Gdm_2_SO_4_ data set were further used to generate a first guess for the respective temperature-dependent concentrations also for the GdmCl data set. In in an iterative process, component spectra were obtained that describe both data sets satisfactorily, such that the effects of the two counterions can be described in a common DNA-structural framework. *Hence, the observed differences in the thermal stability of the Gdm^+^-bound DNA origami will be explained exclusively by altered equilibrium constants between virtually identical structural states in both electrolytes.* Table 1 summarizes the free enthalpy differences between the four components, which approximated the PCA-derived temperature dependencies satisfactorily. Importantly, heat capacity contributions Δ*C*_p_ were allowed to contribute as well (effects were marginal for the numbers set in brackets).

Fig. 8a shows the temperature dependence of the four states derived from PCA (symbols) for Gdm_2_SO_4_ at 4 M Gdm^+^. The salient feature of the model is a sigmoidal temperature-dependent decay of the initial S1-structure of the DNA origami already between 20 and 45 °C in accord with the statistics of structural damage seen in AFM (Fig. 5). The S2 state (“damaged” DNA origami) and a pre-melting intermediate S2’ become populated in parallel up to 55 °C, where the intact DNA origami S1 has fully disappeared and ssDNA not yet formed (*i.e.*, S3 stays at baseline level). Before melting at 64.7 °C, S2 further converts into the pre-melting intermediate S2’ and both structures then decay in synchronicity at *T* > *T*_m_ (this general decay pattern is sketched in a simplified form in the inset of Fig. 8a). The orange-shaded area marks the temperature range with the most prominent CD-spectral change at 240 – 250 nm seen in the TS plot. *Thus, unstacking seen in the CD spectra starts with the onset of the S2 decay at 58 °C.*

In GdmCl at 4 M Gdm^+^, the most intriguing feature is the non-monotonous temperature dependence of the S1-S2 equilibrium below 45 °C, starting with predominantly damaged DNA origami at 20 °C followed by a first inversion of this population at 22 °C and a second at 36 °C (Fig. 8b). The thermodynamic simulation clearly approximates the unexpected temperature dependence of structural damage seen in AFM (Fig. 3). Table 1 shows that the crucial parameter controlling this behavior is the heat capacity change accompanying the S1 → S2 transition. It is about 9-fold larger for GdmCl than for Gdm_2_SO_4_. *Above 45 °C, the general form of the temperature-dependent state population in Gdm_2_SO_4_ is conserved also in GdmCl, i.e., unstacking accompanies the S2 decay.* However, the S2 state starts decaying already at 45 °C, thereby populating the pre-melting intermediate S2′ up to 55 °C. Above this temperature ssDNA formation (S3) starts predominantly from the S2 state already 10 °C below the global *T*_m_. Again, the CD-spectral change in the TS-plot (Fig. 7e) overlaps with the temperature range in which S2 exhibits its largest decay. The simplified sketch of state transitions in Fig. 8b emphasizes the decay of the S2 state into S2′ and S3 already at lower temperature than with sulfate as counteranion.

## 4 Discussion

We have shown that the anions Cl^−^ and SO_4_^2−^ exert surprisingly distinct effects on the thermal stability of DNA origami in the presence of Gdm^+^. Already below the global *T*_m_, stability was modulated differently by the two anions. *The most salient effect was seen with Cl*^−^*, which uncouples base unstacking from base pair opening and increases the heat capacity change upon formation of structures that showed up as “damaged” in the AFM.* A hallmark of cationic interactions with dsDNA is the modulation of the heat capacity changes during DNA melting [49], [50], [51]. However, structural changes in the 23 °C to 40 °C range are unlikely to originate in base pair opening, as there is no rise of the 260 nm absorption below 45 °C. Thus, an entirely different effect on heat capacity must be considered here.

We have addressed the underlying structural changes by CD spectroscopy. Using PCA, we have retrieved anion-independent “component spectra”, which report molecular features of the “intact” (S1) and “damaged” DNA origami (S2) seen in AFM and of a pre-melting state (S2’) and ssDNA (S3) appearing at higher temperatures. These common spectral components reproduced the original CD data sets satisfactorily (Fig. S4) for both Gdm^+^ salts using the temperature-dependent contributions of each state as derived from thermodynamic modeling (Table 1). Fig. S6a shows for comparison the PCA result for DNA origami in the absence of Gdm^+^ salts, where we found that three structural states are sufficient to reproduce the spectral data (thermodynamic parameters in Table S1). Remarkably, the only intermediate state obtained in the absence of Gdm^+^ exhibits the same CD signature as the S2’ state which was independently determined from the data sets with Gdm^+^ (Fig. S6b and c). From this follows that (i) the pre-melting intermediate S2’ is a Gdm^+^-free state populated close to the *T*_m_ and (ii) the “damaged“ S2 structure corresponds to the Gdm^+^-bound DNA state. In fact, the unusually low positive CD amplitude and its frequency shift from 260 nm to 266 nm in the S1 → S2 transition is typical of a strong dehydration of the DNA grooves [52], [53], [54], [55] (water replacement by Gdm^+^) and suggests an increased winding angle in a C-DNA-like state. We think that this change, even by only a fraction of a degree per base, builds up the nanoscopic damage over the 7249 bp seen in AFM images (as a consequence of the decrease in the length of dsDNA by about 0.01 nm per residue with a per residue axial shift for B-DNA ≈ 0.34 nm and for C-DNA ≈ 0.33 nm) [56].

**Fig. 9:**
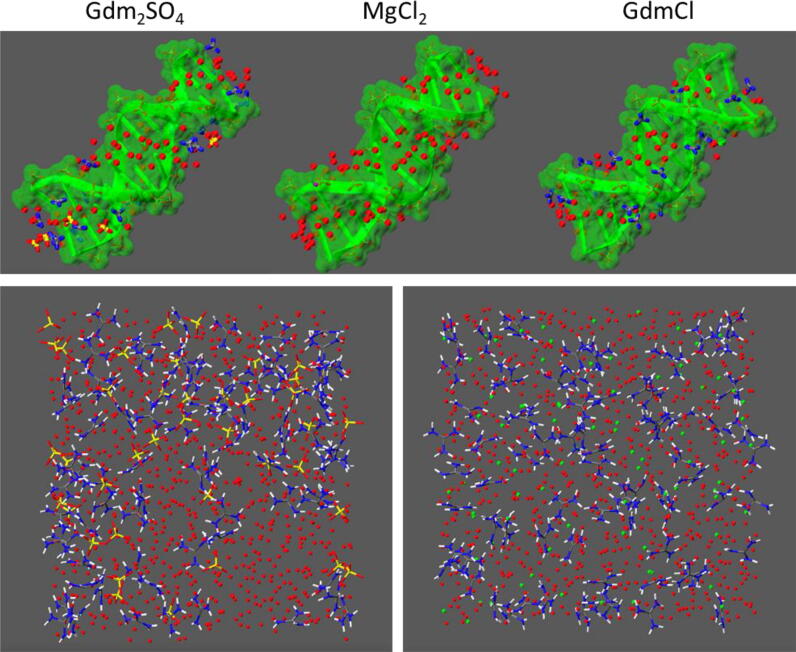
MD simulations of DNA and solvent with Gdm^+^ salts. Upper panel: Representative snapshots of the Drew-Dickerson dodecamer (DNA) in the presence of Gdm_2_SO_4_ (left), Mg^2+^ (middle), and GdmCl (right) from MD trajectories. DNA is depicted in green ribbons with green surface. Water molecules within the DNA grooves (within 5 Å from nitrogen or oxygen atoms of nucleobases) are depicted as red balls. Gdm^+^, Cl^−^, SO_4_^2−^, and Mg^2+^ ions are drawn as ball-and-stick models with Cl^−^ in green, Mg^2+^ in purple, S in yellow, N in blue, O in red, and C in gray. Hydrogen atoms are omitted for clarity. Lower panel: Representative snapshots of 4–5 M Gdm_2_SO_4_ (left) and GdmCl (right) in water from MD trajectories. Color codes as in upper panel with the hydrogen atoms of Gdm are shown in white. In the solution of Gdm_2_SO_4_, ion-free water cavities alternate with extended clusters of paired ions, whereas individual ions are partitioned more evenly in the GdmCl solution. (For interpretation of the references to color in this figure legend, the reader is referred to the web version of this article.)

The spectral analyses explain the S1 and S2 structural features consistently, but how are these linked to different heat capacities? The S1 → S2 transition is driven by the movement of Gdm^+^ into the dsDNA grooves which corresponds to a movement from a weakly water-H-bonded state of Gdm^+^ to a strongly DNA-H-bonded state [21] as Gdm^+^ establishes on average only one H-bond in its inner shell in water clusters larger than eight but can connect to three H-bond acceptors in non-bulk water environments [57]. We think that the different Δ*C*_p_ values are caused by different magnitudes of structural changes in the Gdm^+^-DNA-H-bond network in the S2 state relative to the solvated Gdm^+^ salts. Therefore, MD simulations were carried out with a dsDNA Drew-Dickerson dodecamer in the presence of Gdm^+^ salts. At the same concentration of about 4–5 M Gdm^+^, more cations associate with the DNA grooves in the S2 state when chloride serves as the counteranion (Fig. 9). As a consequence, a highly ordered water cation DNA H-bond network builds up in the minor and major groove of the Gdm^+^-“saturated” S2 state. In contrast, much less Gdm^+^ is bound to the S2 state in the presence of sulfate (Fig. 9), such that the major groove retains more of its normal hydration (Fig. 9). We assign the large heat capacity of Gdm^+^-bound DNA to the strong water-ordering effect of the cation. The concomitant displacement of ordered groove water by ligands has been shown to be entropically favored only in the presence of sufficient bulk-like water [58]. At 4 M Gdm^+^, however, bulk water is barely existent and groove-bound Gdm^+^ increases, rather than decreases the amount of structurally constrained water by both direct H-bonding and by the exposure of its hydrophobic face to the solvent. Similar to the heat capacity increase with protein unfolding [59], these factors increase the heat capacity of the S2 over the Gdm^+^-free S1 state in GdmCl.

The origin of the anion modulation of denaturant binding to DNA lies in the general solvation properties of the two salts, which can be well appreciated in the MD simulations of their solvated states in the absence of DNA. Gdm_2_SO_4_ tends to form ion pairs between Gdm^+^ and SO_4_^2−^ as well as neutral triplets within a shared and highly structured hydration shell due to the strong “oxygen outside” polarization of the sulfate ion hydration [60]. This leads to a microscopic demixing of the solution into sequestered close to electrically neutral Gdm^+^-sulfate networks on the one hand and of ion-free water domains on the other (Fig. 9). The release of water structural constraints by removing Gdm^+^ from these clusters seems to be offset by a similar increase of such constraints in the DNA-bound state of Gdm^+^.

In contrast, GdmCl exhibits less ion pairing, leading to efficient association of the uncorrelated Gdm^+^ cation with the negatively charged DNA in the first place. Secondly, the six-fold coordinated Cl^−^ ion preserves the average tetrahedral water network of bulk water [35]. Thereby, less water structure needs to be broken in a GdmCl solution than is formed upon Gdm^+^ binding to DNA, hence the larger Δ*C*_p_ of the S1 → S2 transition. Ion pairing correlates with low activity of the respective salts, which again is considered to be the case when the hydration shells of the paired cation and anion are similar. Comparable to the salt-dependent structural transitions observed here, the folding of RNA has been shown to be differently affected by a given cation when it is paired with a similarly-sized “matching” (low activity salt) or a differently sized “non-matching” anion (high activity salt) [61]. Gdm^+^ with sulfate and chloride forms such a matching and non-matching pair, respectively. We have shown that competing ion and co-solute hydration patterns lie behind this phenomenological distinction. The DNA origami as a nM cosolute in 4 M Gdm^+^ denaturant solution appears to sensitively transduce these molecular processes into nanoscopic structural transitions and reveals hydration-dependent heat capacity changes as the key thermodynamic parameter that regulates the temperature-dependent stability of a supramolecular DNA assembly in a denaturing environment.

## 5 Conclusion

We have shown that DNA origami thermally denature in a complicated manner. At ambient temperatures, heat capacity changes determine the relative amount of an “intact” structure in the presence of Gdm^+^ salts. This explains the counter-intuitive rise and decline of “intact” DNA origami with temperature in the 23 °C to 45 °C range as the free enthalpy of reaction is not a monotonous function of temperature anymore. The heat capacity change between “intact” and “damaged” structures is significantly enhanced by the presence of GdmCl but not Gdm_2_SO_4_. It appears to originate in the more water-like and less charged hydration shell structures in GdmCl and the lack of ion pairing as compared to Gdm_2_SO_4_. Thereby, under the overall water-limited solvation conditions at 4 M Gdm^+^, the transfer of the denaturing cation from solvent to DNA upon heating increases the number of ordered low entropy water networks around the DNA origami much more for the chloride than the sulfate counterion. Finally, the main DNA melting transition in the Gdm^+^ salts transits through a Gdm^+^-dissociated pre-melting intermediate, which exhibits hydrated DNA and is structurally equivalent with the “naturally” occurring pre-melting intermediate observed also in the absence of Gdm^+^.

Our data suggest that the supramolecular structure of a DNA origami amplifies subtle steric effects by their accumulation over the large number of linked dsDNA segments. While similar amplification effects have recently been found also in DNA origami degradation by UV irradiation [62] and reactive oxygen species [63], this is the first time that such a behavior could be observed not only at the nanostructural but also at the molecular level. Therefore, in combination with the thermodynamic analysis, geometric, energetic, and hydration effects accompanying DNA-ligand interactions may find a novel sensitive nanoscopic and thermodynamic readout.

## Declaration of Competing Interest

The authors declare that they have no known competing financial interests or personal relationships that could have appeared to influence the work reported in this paper.
