# Supplementary material for: Anion-specific structure and stability of guanidinium-bound DNA origami
Source: Comput Struct Biotechnol J. 2022 May 23;20:2611–23. doi: 10.1016/j.csbj.2022.05.037 (PMC9163702; doi:10.1016/j.csbj.2022.05.037)
Supplement: Supplementary Data 1 [file mmc1.pdf]

Supplementary Material

Anion-specific structure and stability of guanidinium-bound DNA origami

Marcel Hanke, Daniel Dornbusch, Christoph Hadlich, Andre Rossberg, Niklas Hansen, Guido Grundmeier, Satoru Tsushima, Adrian Keller, and Karim Fahmy**

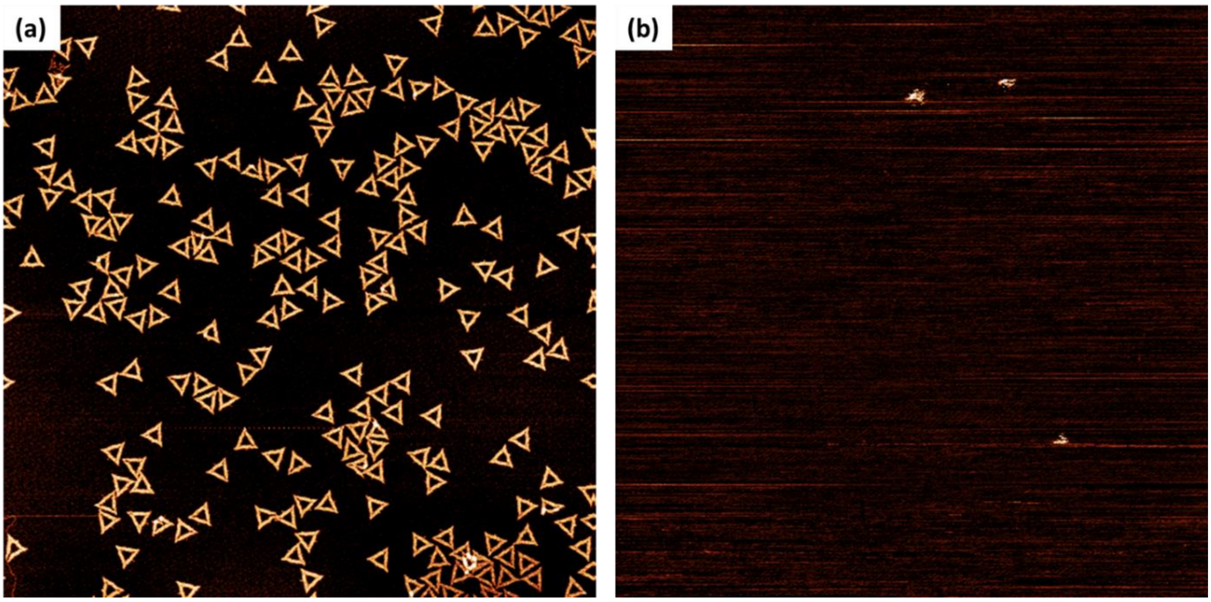

Fig. S1 AFM images of DNA origami triangles deposited on mica. (a) Imaged in air after drying. (b) The same sample subsequently imaged in Tris/$MgAc_2$ buffer with 6 M Gdm_2SO_4. The images have a size of 3 x 3 μm^2 and a z-range of 2 nm.

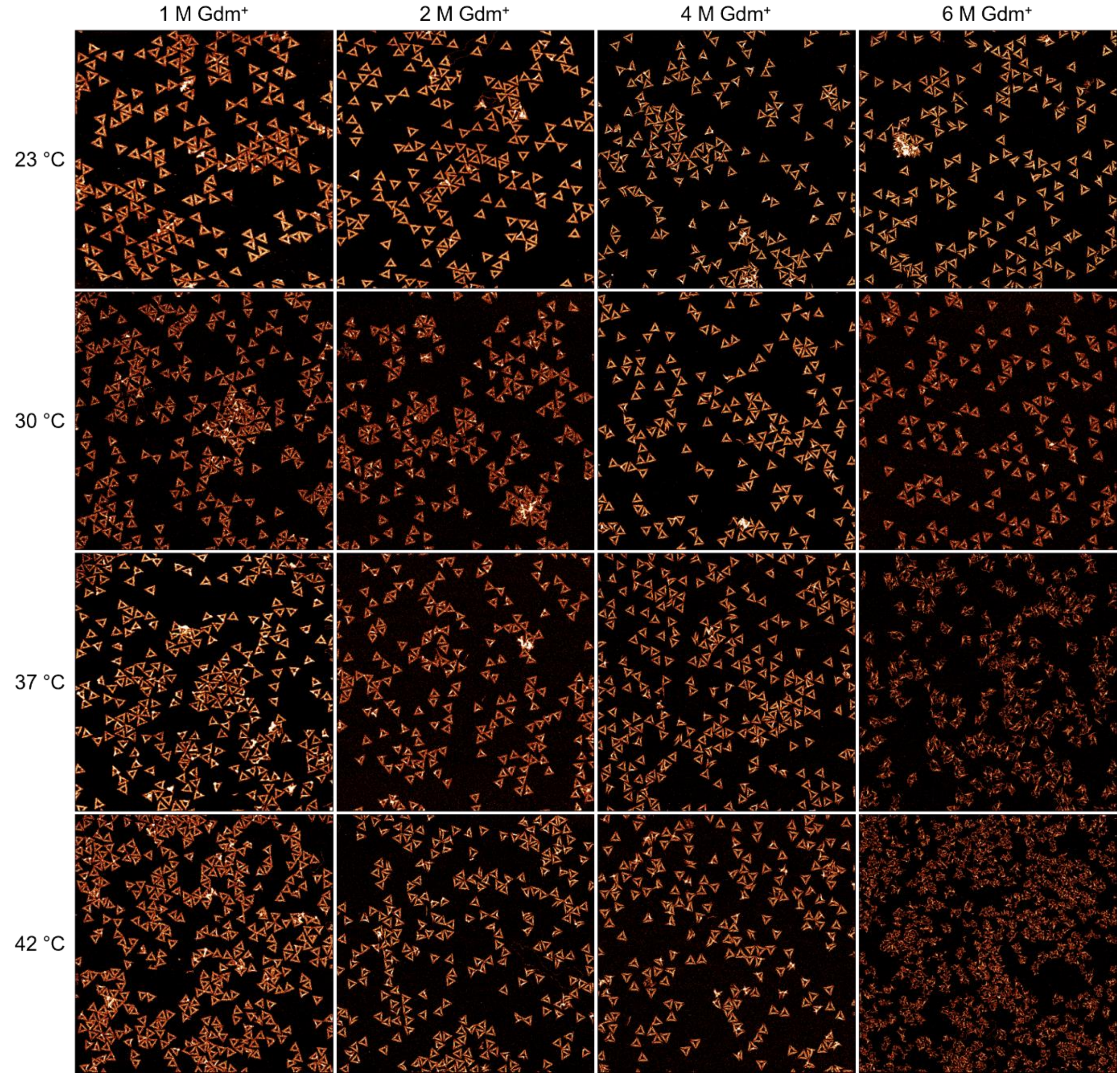

Fig. S2 AFM images of DNA origami triangles deposited on mica after 1 h incubation in GdmCl at different Gdm^{+} concentrations and temperatures. All images have a size of 3 x 3 μm^2 and a z-range of 2 nm.

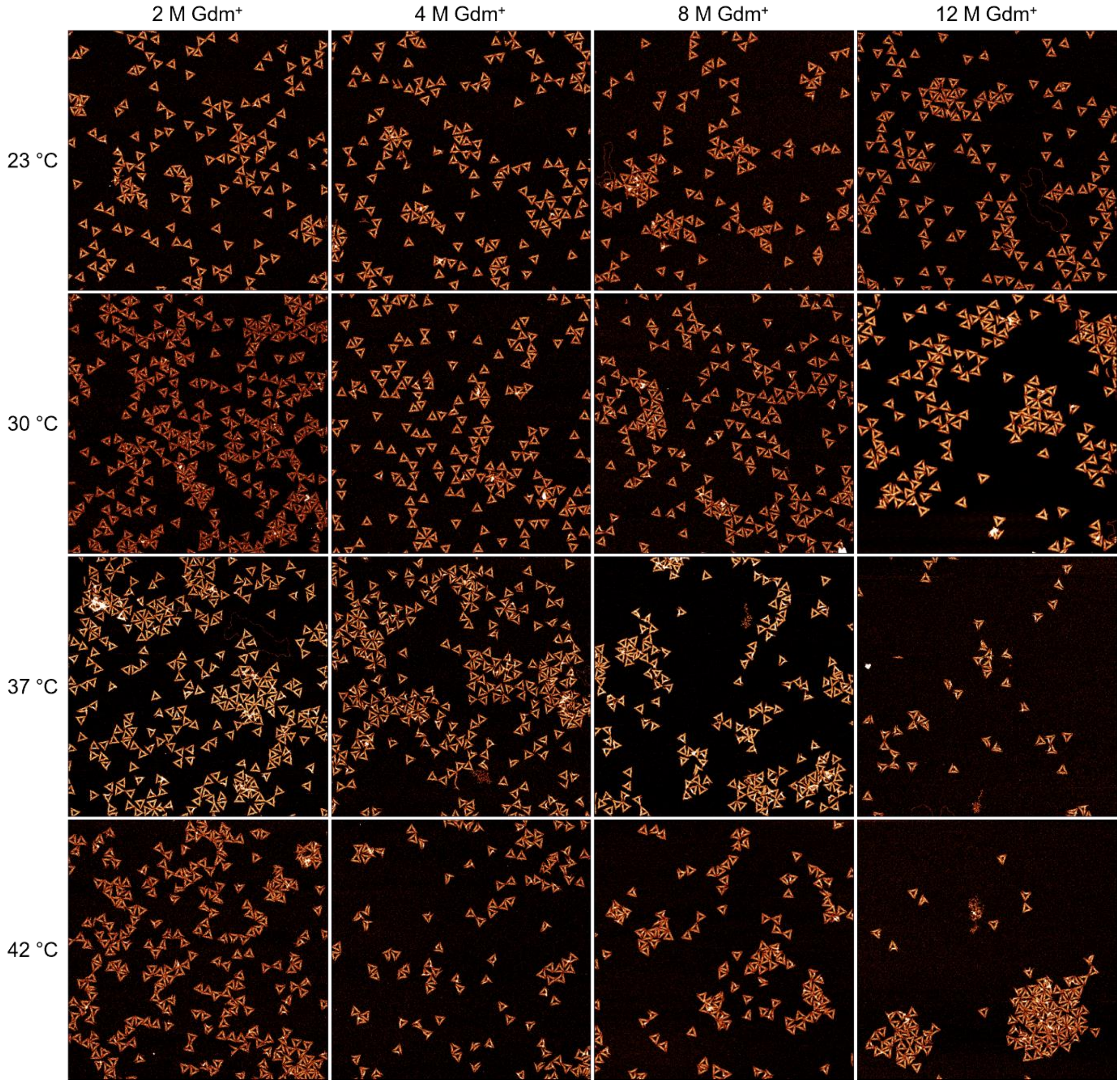

Fig. S3 AFM images of DNA origami triangles deposited on mica after 1 h incubation in Gdm_2SO_4 at different Gdm^+ concentrations and temperatures. All images have a size of 3 x 3 μm^2 and a z-range of 2 nm.

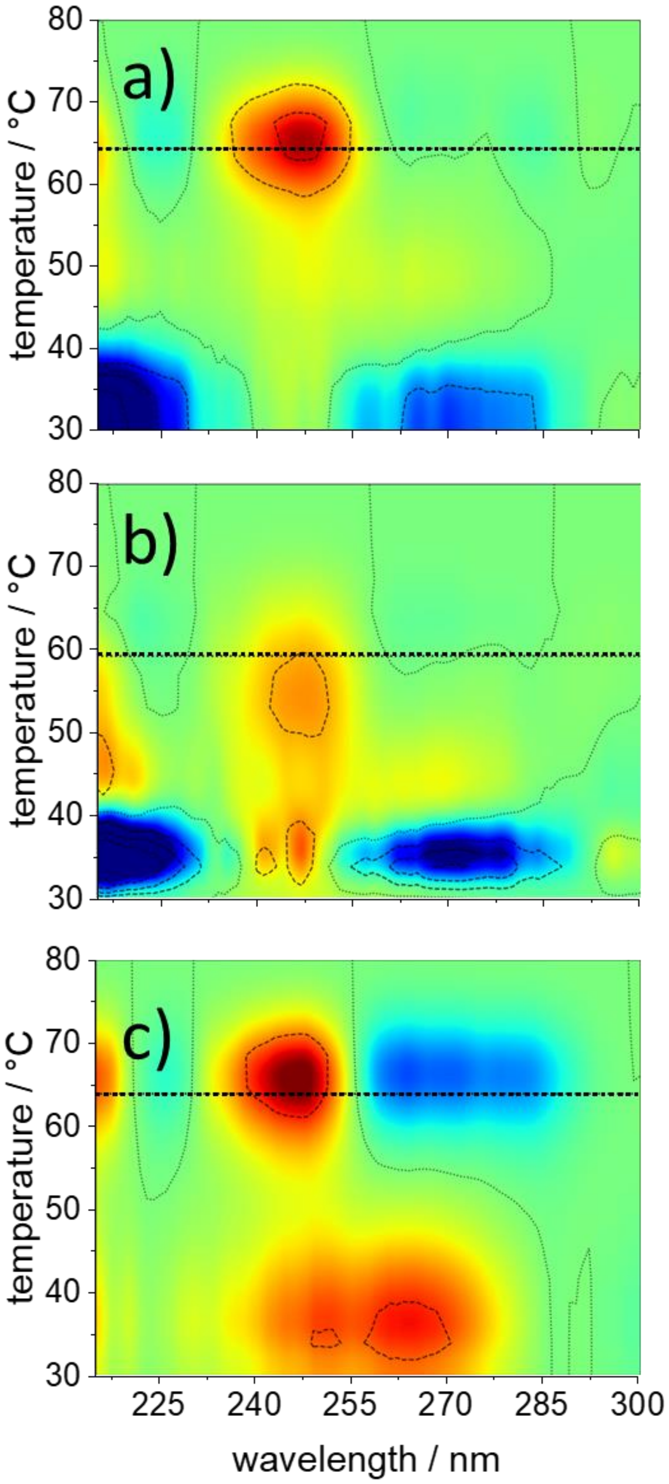

Fig. S4 Reproduction of the original temperature sensitivity of the CD data sets for Gdm_2SO_4 (a) GdmCl (b) and the control without Gdm^+ salt (c) using the component spectra for the structural states S1, S2, S2', and S3 (Figure S6c) and the corresponding concentrations given by the thermodynamic model (Table 1, and Figure 8).

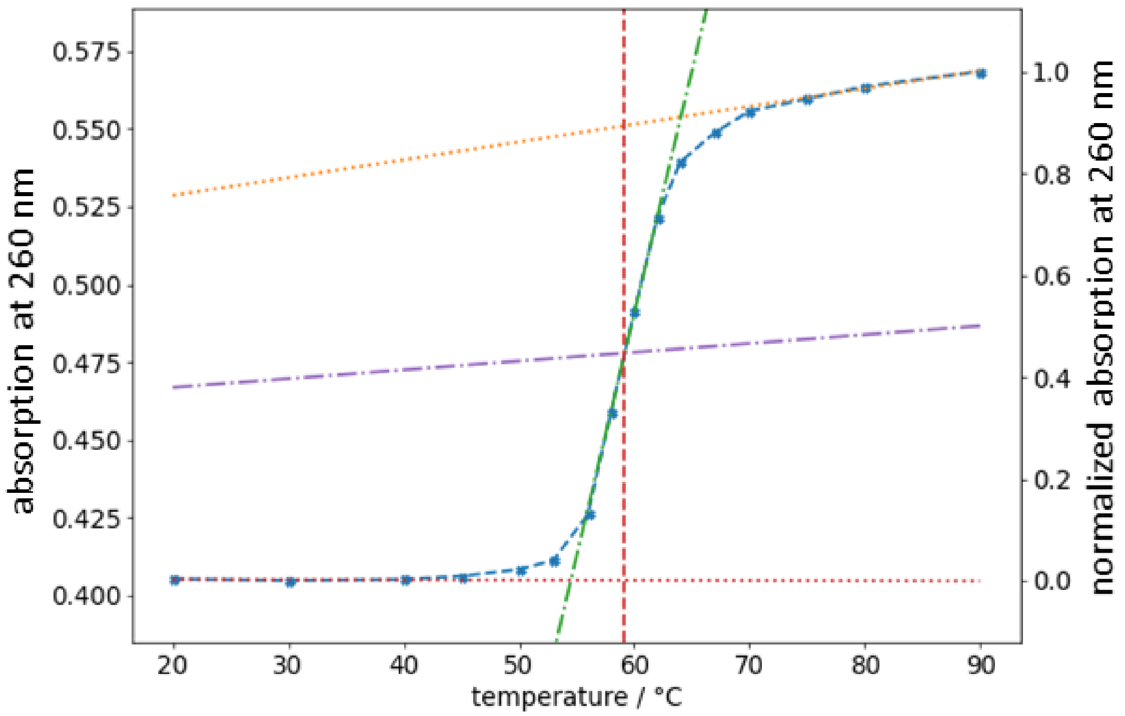

Fig. S5 Determination of the gobal DNA origami melting temperature T_m. The method is exemplified here for 4 M GdmCl. The UV absorption of the three initial and final data points was linearized (red and orange, dot) and an averaged linear equation determined (violet, dash-dot). The data pairs lying closest above and below the averaged line were approximated by another straight line (green, dash-dot) and the T_m (red, dash) interpolated as their crossing point. The 260 nm absorption was measured during the recording of the CD spectra evaluated in Figure 7e and 8b using the normalized absorption as the relative concentration of ssDNA.

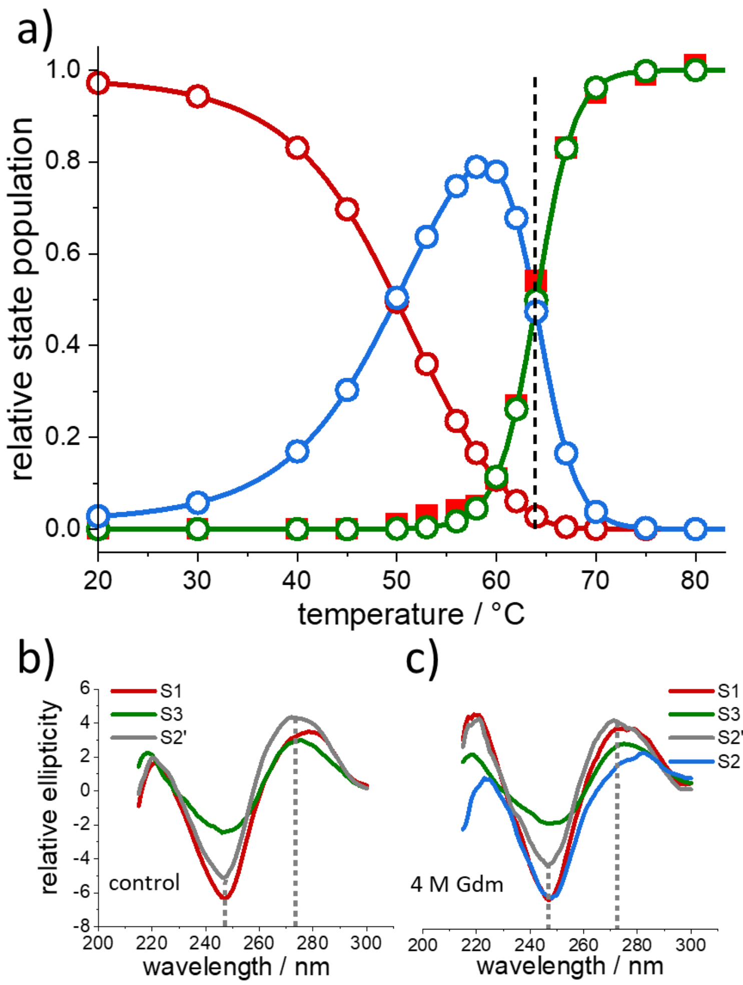

Fig. S6 Factor analysis and thermodynamic modelling of the thermal denaturation of Gdm^+-free DNA origami. (a) Open circles: state population from PCA; filled red squares: normalized 260 nm absorption change; solid lines: state population from thermodynamic model (Table 1). (b) CD-spectra from PCA shown in (a). (c) CD spectra obtained from the PCA of the Gdm^+-containing samples (averages of the two respective component spectra obtained with the Gdm_2SO_4 and GdmCl data sets). Color codes as in Figure 8.

Table S1 Thermodynamic parameters for state transitions in the Gdm^+-free DNA origami in the presence of Mg^{2+}.

	S1 → S2’	S2‘ → S3
ΔH (kJ)	155	480
ΔS (kJ/K)	2,505	7,511
ΔC_p (kJ/K)	4000	(10,000)
T_m (°C)	49.9	63.9
